# Adherence therapy versus routine psychiatric care for people with schizophrenia spectrum disorders: a randomised controlled trial

**DOI:** 10.1186/s12888-016-0744-6

**Published:** 2016-02-25

**Authors:** Wai Tong Chien, Jolene Mui, Richard Gray, Eric Cheung

**Affiliations:** School of Nursing, Faculty of Health and Social Sciences, The Hong Kong Polytechnic University, Hung Hom, Kowloon, Hong Kong, S.A.R. China; Castle Peak Hospital, Tuen Mun, New Territories, Hong Kong, S.A.R. China; Hamad Medical Corporation, P.O. Box 3050, Doha, Qatar

**Keywords:** Adherence therapy, Antipsychotics, Randomised controlled trial, Motivational interviewing, Schizophrenia, Insight into treatment

## Abstract

**Background:**

Current practice guidelines for schizophrenia care recommend that antipsychotic medication is essential for patients’ long-term maintenance treatment but their non-adherence to this medication is still a main obstacle to relapse prevention. This study evaluated the effects of a motivational-interviewing-based adherence therapy for people with schizophrenia spectrum disorders.

**Methods:**

This randomised controlled trial was conducted with 134 outpatients with schizophrenia spectrum disorders; 67 of them received a six-session adherence therapy (in addition to usual care) and 67 received usual psychiatric care alone. Participants’ outcome measures included symptom severity, medication adherence, hospitalisation rates, insight into illness/treatment, and functioning.

**Results:**

The adherence therapy group reported significantly greater improvements in symptom severity (*p* < 0.003), insight into illness/treatment (*p* < 0.001), functioning (*p* < 0.005), duration of re-hospitalisations (*p* < 0.005), and medication adherence (*p* < 0.005) over 18 months follow-up, when compared with usual care alone.

**Conclusions:**

Motivational-interviewing-based adherence therapy can be an effective approach to treatment for people with early stage of schizophrenia who poorly adhere to medication regimen.

**Trial registration:**

ClinicalTrials.gov NCT01780116, registration date January 29, 2013.

## Background

Schizophrenia and its spectrum disorders is a major group of serious mental illnesses that induces disabling residual and remitted symptoms and high relapse rates in psychiatric rehabilitation globally [1]. While current guidelines for schizophrenia care recommend antipsychotic medication to be essential for long-term maintenance treatment, non-adherence to this medication is found to be a predictor factor for re-hospitalisation and relapse [1, 2]. It is also recommended that mental healthcare interventions and services should explicitly direct healthcare providers to ensure that early psychosis intervention teams are in place and provide appropriate psychosocial interventions for these people within the ‘critical period’ (i.e., the first 3 years of illness), which can address their specific needs for better engaging and retaining in the services/interventions provided, enhanced insight into the illness and its treatment and optimal self-management of the illness [3]. Therefore, it can facilitate early recovery and prevent their progression to chronic illness.

Systematic reviews on clinical trials of treatments in psychosis, particularly those with chronic psychosis, suggest that these patients’ adherence to oral antipsychotics are generally poor with average adherence rates of 30–50 % [4, 5]. Despite the advent of new (atypical) antipsychotics with less side-effects, some side-effects of these novel drugs such as tardive dysknesia and obesity are severe. While there has been increasing number of patients using these atypical antipsychotics, there is little evidence that progress has been made on increasing these patients’ medication adherence. Poor medication and other treatment compliance may then result in frequent recurrences and high relapses from schizophrenia, which would much increase the medical costs and burden of society, for example, ranging from U.S. dollars of 4–12 billion and contributing to 2–5 % of the total medical or Medicare costs of developed countries [1, 5]. Early intervention for people with first-episode or recent onset psychosis involving education and experiential learning in dimensions of awareness of illness, relabeling of symptoms and need for treatment is considered essential to enhance their motivation to engage in psychosocial intervention and rehabilitation programmes [3]. Adherence therapy may be useful for these peoples with early psychosis to improve their insight to the illness and its medication/treatments, and enhance their motivation and self-efficacy in self-management of their illness and relapse prevention [6, 7].

‘Adherence’, sometimes interchangeably with ‘compliance’, means that a client accepts the advice of healthcare professionals to take medication according to a medical prescription, and it reflects the client’s perspective regarding the importance and purpose of taking the prescribed medication [4, 6]. Nevertheless, the effects of psycho-education and other models of psychosocial intervention on adherence to medication in schizophrenia are multifaceted and complex, with modest and inconsistent effects on successfully improving patients’ treatment adherence, functioning and/or other psychosocial outcomes [2, 5, 6]. For instance, psycho-education programs for people with psychotic disorders aimed to enhance knowledge about mental illness and its medications/treatments; however, the findings of most recent studies were non-significant on promoting positive attitudinal and behavioural changes in these patients’ treatment adherence and improve the service utilization and re-hospitalisation rate [3, 7–9].

While poor insight has shown to be associated with poor outcome in schizophrenia [10], insight is regarded as a multi-dimensional construct with at least three interrelated components, including awareness of having a mental illness, understanding the need for treatment and its compliance, and the ability to re-evaluate and re-label unusual events as pathological [11]. These components of insight appear to be distinct constructs with different neurocognitive and psychosocial correlates [12, 13]. Acute and severe psychopathology (e.g., suspicions and delusional beliefs) and any delay in seeking treatment during the early stages of schizophrenia associated with insight into the illness can render it difficult for prompt and efficacious interventions and understanding about the significance of medication adherence, thus reducing the likelihood of their adherence behaviours [7, 9]. Patients’ insight into treatment can be associated with their negative symptoms and internalized stigma.

This may be because similar to other chronic illnesses, these interventions have inadequately focused on facilitating these patients to accept their illness and its treatment and/or resolve their resistance or ambivalence to changing their lifestyle and behaviours, as required by the treatment regimen [2, 7]. Most approaches to psychosocial intervention in schizophrenia mainly provide knowledge of the illness and its treatment and prognosis, perceived (or experienced) stigma, coping and behavioural adjustments, and therapeutic alliance with professionals. However as suggested by recent expert consensus guidelines for schizophrenia treatment [1, 6], motivational interviewing (MI) technique has recently been adopted in adherence therapy (AT) for people with schizophrenia to enhance their adherence to medication by helping them understand and accept their medication receiving and cope with the life situations concerning the adverse effects of the medication taken. Unlike most of the other psycho-educational and behavioural approaches to medication adherence, MI is a goal-directed, patient-centred interventional style, which specifically works on facilitating and engaging intrinsic motivation within individual patients and helps them explore and resolve ambivalence to an adherence behaviour, for empowering them to consider making changes in such non-adherence [7, 14]. The MI-based intervention has demonstrated preliminary positive evidence on reducing psychotic symptoms and relapse rate in a few European countries and Thailand [7–10]. Therefore, this study aimed to evaluate the effect of adherence therapy for Chinese people with schizophrenia and its subtypes on symptom severity, medication adherence, re-hospitalization rate, functioning, and insight into illness/treatment over a 18-month follow-up, when compared with those receiving treatment as usual (TAU). It was hypothesised that that the AT group would demonstrate significantly greater improvements on symptom severity and other clinical outcomes (e.g., medication adherence and re-hospitalization rates, and insight into illness/treatment) at immediately and over 18 months after completion of the intervention, than the TAU group.

## Methods

This was a single-blind randomised controlled trial of a motivational interviewing-based adherence therapy for outpatients with schizophrenia spectrum disorders, using a repeated-measures control group design and an intention-to-treat basis. The trial was undertaken in Hong Kong between December 2012 and May 2015 and all subjects were assessed by a psychiatrist to be mentally competent to follow the instructions of the intervention used and followed up over 18 months (Period: May 2012-December 2014), respective of whether they completed the intervention or not.

### Participants and settings

**Fig. 1:**
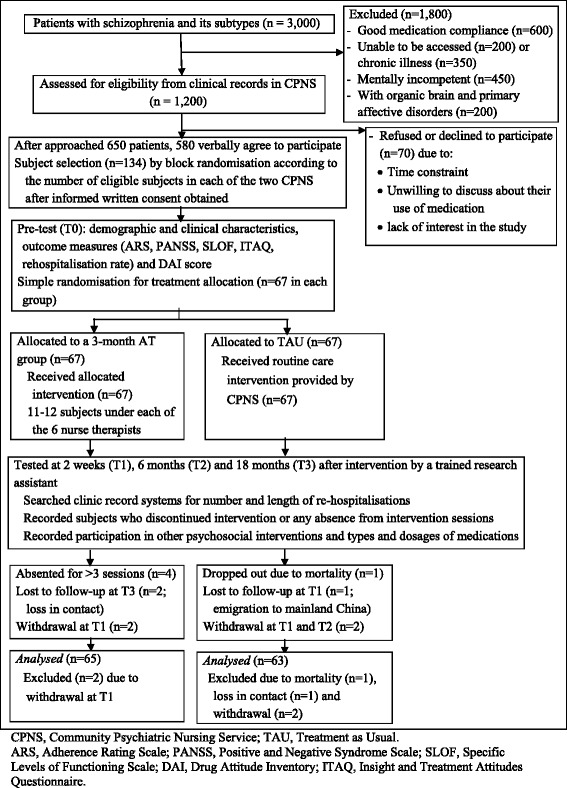
This figure indicates a flow diagram of the controlled trial procedure. One hundred and thirty-four out of 650 approached patients with schizophrenia spectrum disorders were recruited from two community psychiatric nursing service units (i.e., 67 participants in each setting). After informed consent and baseline measurement, they were randomly assigned into either adherence therapy (plus usual care, *n* = 67) or treatment as usual alone (*n* = 67) group. Following 3-month intervention, the participants completed the post-test outcome measurements at 2 weeks, 6 months and 18 months follow-up. With an attrition of totally 6 participants, outcome data of 65 in adherence therapy and 63 in treatment as usual group were finally analysed and compared

The controlled trial was conducted at two Community Psychiatric Nursing Services (CPNS) in the two largest geographical regions of Hong Kong (i.e., the New Territories and Kowloon). There were 3000 patients with schizophrenia and its subtypes such as schizoaffective and schizophreniform disorders; of which, 1200 were eligible participants (40 % of the total patient population) who met the study criteria at below and were accessible. The main reasons for those excluded from this study (as shown in Fig. 1) included: good medication compliance (*n* = 600), unable to be accessed (*n* = 200), having chronic physical illness or cognitive impairment (*n* = 350), and mentally incompetent to participate (*n* = 450). From 1200 eligible participants with schizophrenia spectrum disorders, 650 (54 %) could be contacted and agreed to participate and 134 were recruited after obtained their informed written consent by a research assistant. There were 70 patients (11 %) approached but refused to participate mainly due to lack of time or unwillingness to discuss their medication adherence. Sixty-seven participants per CPNS were selected randomly from each of the two patient lists (in alphabetical order with their names), using computer-generated random numbers prepared by an independent statistician. After completed the baseline measures during home visit, 134 participants were assigned randomly by the research assistant into either AT or TAU (*n* = 67 in each group) by drawing a labelled card (1 = ‘AT’; 2 = ‘TAU’) from an opaque envelope. The participant list and intervention assignment were concealed to the researchers, assessors and outpatient clinic until all data entries were completed.

Patients with schizophrenia spectrum disorders (according to DSM-IV-TR criteria) [15] from the two CPNS were included if they were: (a) aged 18–64 years, Hong Kong residents speaking in Mandarin/Cantonese; (b) within 3 years of the illness; and (c) poorly adhered to antipsychotic medication as indicated by the Drug Attitude Inventory score of 11 or below [7], and/or recent history of non-adherence to medication (i.e., any cessation of oral antipsychotics associated with psychiatric admission for ≥1 per month, or with ≥3 missed doses/week in the past 3 months [5, 7, 9], as indicated in their outpatient or CPNS records). Those were excluded if they had: (a) regular medication of depot/intramuscular injection(s) only; (b) co-morbidity of learning disability, organic brain disease and/or a clinically significant medical disease; (c) already participated in any medication management programme; and/or (d) supervised medication taking by health care staff.

Sample size estimation was based on two similar clinical trials and our pilot study of adherence therapy, in which symptom severity and/or medication adherence were the primary outcomes [8, 9, 16]. A random sample of 134 (*n* = 67 per group) was required to detect significant differences on the two outcomes between two groups at an effect size of 0.54 (0.58 for symptoms and 0.50 for adherence) [9, 16], at *p* = 0.05 (two-sided) and study power of 0.80, with a 20 % of expected attrition [17].

### Procedure

Ethics approval of this trial was granted by the Human Subjects Research Ethics Sub-Committee of The Hong Kong Polytechnic University and the Hospital Cluster Research Ethics Committees of the hospitals (KCH and CPH) governing the two CPNS. A flow diagram of the study procedure is attached in Fig. 1 according to the latest CONSORT statement [18]. All participants were fully explained about the purpose and procedure of the study, as well as the confidentiality of data and their right to withdraw from the study at any time, and then asked for written consent. With their written consent, the participants were asked to complete the outcome measures and demographic and clinical data at recruitment (T0) and 2 weeks (T1), 6 months (T2) and 18 months (T3) after completed 3-month intervention. Patients’ re-hospitalisations (and its nature such as voluntary and compulsory) and duration of illness were checked and confirmed with the patient records in the CPNS.

### Measures

The outcome measures at T0-T3 included: the primary outcomes, consisting of Adherence Rating Scale (ARS) and Positive and Negative Syndrome Scale (PANSS); and the secondary outcomes, including Insight and Treatment Attitudes Questionnaire (ITAQ), Specific Level of Functioning Scale (SLOF), and number and length (days) of psychiatric hospitalizations, over the past 4 months. These scales showed very good internal consistency among the participants at baseline measurement in this study (i.e., Cronbach’s alphas were 0.89 for ARS, 0.91 for PANSS, 0.88 for ITAQ, and 0.93 for SLOF). Total number and duration (days) of psychiatric re-hospitalisations of each participant were self-reported by the participants and then checked against the outpatient clinic/CPNS records at pre-test and three post-tests. Patients’ demographic and clinical data (e.g., gender, age, dosage of antipsychotic medication in terms of haloperidol equivalents [19], and duration of illness) were collected at recruitment.

The single-item ARS developed by Staring et al. [10] was a simple, user-friendly and valid measure to assess level of medication adherence; whereas, pill counts and urine tests have recently been recognised for its potential inaccuracy and time constraints for estimation of usage of antipsychotic medication [7, 9]. Items of the ARS were rated on a 5-point Likert scale (1 = total non-adherence, 2 = poor adherence, 3 = inadequate adherence, 4 = fair adherence, and 5 = good adherence) by the research assistant and participants’ case manager (community psychiatric nurse), independently; and their ratings were finalised in consensus agreement. The scale demonstrated excellent inter-rater reliability (92–100 % agreement) and content validity in people with psychotic disorders [16, 20].

The 30-item PANSS assessed the severity of psychotic symptoms on three subscales, including positive symptoms (7 items), negative symptoms (7 items) and general psychopathology (16 items) [21]. Items were scored on an 8-point Likert scale (from 1 = absent to 7 = extreme). The scale indicated a high concurrent validity with the Brief Psychiatric Rating Scale (Pearson’s *r* = 0.85–0.90) and satisfactory test-retest reliability (intra-class correlation = 0.85–0.90) and internal consistency (Cronbach’s α = 0.88–0.91) in patients with serious mental illness [21].

The 11-item ITAQ assessed patients’ insight into their illness and needs for treatment [22]. Items were rated on a 3-point Likert scale (0 = no insight; 1 = partial insight and 2 = good insight); the higher its score, the better was a person’s insight into the illness and receiving its treatments. The Chinese version indicated satisfactory internal consistency (Cronbach’s α = 0.82), inter-rater reliability (intra-class correlation = 0.82), and concurrent validity with mental status and psychopathology (Pearson’s *r* = 0.56 and 0.60, *p* = 0.001) measurements in Chinese patients with schizophrenia [16, 23].

The 43-item SLOF assessed three functional domains of patients with schizophrenia, including self care and maintenance (12 items), social functioning (14 items) and community living skills (17 items) [24]. Its items were rated on a 5-point Likert scale (from 1 = totally dependent to 5 = highly self-sufficient). The Chinese version demonstrated satisfactory content validity, test-retest reliability (intra-class correlation = 0.80), and internal consistency (Cronbach’s α = 0.88–0.96) in Chinese patients with schizophrenia [16, 23].

### Adherence therapy (AT)

Patients (*n* = 67) received a 12-week (six 2-h sessions every 2 weeks) AT modified from Gray et al.’s [7] medication adherence programmes, in addition to usual CPNS. Most concepts, topics and content of Gray et al.’s 8-session AT, which was based on Kemp et al.’s [25] compliance therapy, were adopted, including the approach and principles of motivational interviewing (MI) with non-confrontational technique, re-examining and improving knowledge, attitude (ambivalence) and barriers to medication adherence, planning for problem-solving and actions for changes in adherence behaviour, rationalising patient’s beliefs and concerns, and relapse prevention. The AT conducted by a trained community psychiatric nurse (nurse therapist) during home visit was based on the principles of MI, involving cognitive, motivational, insight-inducing, and behavioural training, which are viewed as particularly useful for people with addictive and resistant behaviours or ambivalent to changing their attitude/behaviour towards psychiatric treatment [7]. The nurse therapists used the five principles of MI with a non-confrontational approach, including expressing empathy, developing discrepancy, avoiding from argumentation, rolling with resistance, and supporting self-efficacy [25], for discussing their attitudes and beliefs towards their illness and medication adherence. While it was considerably more difficult to discuss issues of motivation in patients with severe psychotic (positive and negative) symptoms, MI combined with principles of cognitive therapy (e.g., awareness and acceptance of experiences, problem-solving and coping skills training, and behavioural rehearsals of adherence) was found useful to focus on particular consequences of illness and treatment behaviours on individual patients, thus engaging them in resolving their ambivalence to their illness-related and life problems [9, 16].

The AT consisted of three phases (Table 1), adopting a few strategies to address Chinese cultural tenets (i.e., cultivating an open and accepting mode of responses and expression of feelings, resolving strong self-centredness, discussing perceived social stigma, and encouraging family support and collectivism) [16]. Phase 1 (one session) of AT aimed to engage participants in addressing their needs and concerns in medication adherence, leading them to set goals and action for change in non-adherence. Phase 2 (two sessions) focused on education about the illness and its treatment, then explored patients’ strengths and barriers to medication adherence and facilitated them to recognise main barriers to their adherence such as perceived stigma, undesirable side-effects of medication and inadequate social support, and finally, developed coping strategies in taking medication over a long term. Phase 3 (three sessions) aimed to rationalise patients’ beliefs and concerns about medication management, resolve difficulties in adhering to medication regimen and improve social network and relationship, thus empowering relapse prevention and better integration into the community.

**Table 1:** Motivational interviewing-based adherence therapy for people with schizophrenia

Phase/Session	Interventions	Main assignments
Phase 1 (1 session)	Purposes:	Reviewing antipsychotic medication use and the impacts of psychotic symptoms on medication (and treatment) adherence, the desired and unwanted effects of medication, anti-psychotic side-effects, and attitude and satisfaction with medication taking. Examining and addressing beliefs and concerns towards adherence, and plan for problem-solving. *Homework assignment*: Weekly record of adherent behaviour and reasons for adherence and/or inadequately or fully non-adherence.
	(1) To help participants review their past and present states of taking antipsychotics; and (2) To assess knowledge, attitude and barriers to medication adherence and plan for problem-solving and improving adherence behaviour using a standard assessment form. Participants identify the present beliefs and concerns, benefits and barriers related to medication and rated the level of distress (i.e., 1–10) attached to each of the main side-effects. Family members are asked for giving their opinions and attitudes towards medication taking by their relative with schizophrenia. Participants are asked to do home assignment by recording weekly adherent behaviours and both they themselves and nurse therapist would keep documentation for records and reviews. The therapist makes an attempt to link medication cessation with relapse. Negative treatment experiences and high levels of distress regarding side-effects are acknowledged and discussed. Denial of need for treatment is met with gentle enquiries into the ensuring social/family/lifestyle disruptions.	
Phase 2 (2 sessions)	Purposes:	Revisiting and revising previous goals or add new ones, and their actions. Recognising factors that may lead to poor adherence, and develop coping strategies to reduce urges for non-adherence *Homework assignment:* Practicing new actions for maintaining or enhancing adherence. Weekly record of adherent behaviours and reasons for adherence or non-adherence to medication.
	(1) To educate about mental illness and its treatment and care required; (2) To review the goals, actions and adherence records of the last two weeks; and (3) To identify barriers to medication adherence and develop coping strategies, immediate and longer-term goals/actions. Participants’ misconceptions about symptoms and side-effects antipsychotic medication will be clarified. The tendency to stop medication whenever the participants feel well is to be discussed, and their meanings attached to medication are explored, that is, an identity as a ‘sick person’. Participants are asked to weigh up their benefits and drawbacks of treatment and the nurse therapist ‘home in’ on the benefits, especially when they emerge spontaneously. Symptoms reported by the participants are fed back as their needs (‘symptoms’) for treatment.	
Phase 3 (3 sessions)	Purposes:	Evaluation of the progress of medication adherence with each participant and his/her change in beliefs/insight into illness and treatment during session 5. Making future plan with participants to continue self-monitoring of adherence and its contractual agreement; and clarifications of means of support from CPNS, family and services. *Homework assignment:* Weekly record of medication behaviours and reasons for adherence or non-adherence. *Risk assessment* for relapse prevention and a list of risk factors identified and recorded.
	(1) To rationalise participant’s beliefs and concerns and to prevent relapse; (2) To manage social stigma and enhance social support. Participants are facilitated to identify the characteristics of prodromal symptoms and the importance of early intervention to prevent a full-blown episode. In sessions 5 and 6, the nurse therapist use normalising rationale to deal with stigma towards the illness/medication; suggest analogy with physical illness requiring maintenance treatment; and highlight illness prevalence with examples of ex-patients who have been successful in coping with difficulties as theirs. Participants reframe medication use by participants as a freely chosen strategy to enhance control of quality of life and use metaphors of medication as ‘insurance policy’- staying well. Future plan and contractual agreement are made to continuously monitor medication adherence and supporting resources from CPNS, family and other mental healthcare services are clarified.	

*CPNS* Community Psychiatric Nursing Service

The AT was conducted by six community psychiatric nurses (i.e., three from each CPNS) who received two full-day training and supervised practices on four patients based on the AT instructor programme [7, 8]; and each of them was responsible for 11–12 participants. The consistency and competency of their implementation of the AT (i.e., over 90 % of the items rated as ‘competent’) was confirmed, using a validated AT competency scale [9]. Two sessions of each therapist were randomly selected, audio-taped and assessed by two raters (first author and one researcher) independently to monitor the fidelity and competency of implementation of AT according to the treatment protocol. The fidelity of their intervention implementation ranged between 91–99 % of the items required and 92–97 % of them also rated as ‘competent’ (median = 96 %).

### Treatment as usual (TAU)

The TAU group received routine treatment and community psychiatric nursing service, which were similar between the two CPNS. The TAU consisted of psychiatric consultation and treatment by psychiatrist, home visits, mental health assessment and brief education on treatment and medication by community psychiatric nurse every 4–6 weeks, and healthcare, social welfare and financial aids by medical social worker. Clinical psychologist would also be consulted for counselling as needed.

### Data analyses

Demographic and outcome data of the two study groups (AT and TAU) were analysed on an intention-to-treat basis using the IBM’s SPSS version 20.0. A Goodness of Fit Chi-square (for categorical data) and independent-sample *t*-test (for interval/ratio data) were used to test the heterogeneity of the study groups or settings in terms of demographic characteristics and outcome measures at baseline (T0). With too much violations of assumptions of linearity and homogeneity of variance-covariance, and multi-collinearity for multivariate analyses of variance [17], repeated-measures analysis of variance (ANOVA) tests were performed for most of the outcome variables (ITAQ, PANSS, SLOF, ARS, and length of re-hospitalisations), and Kruskal-Wallis test for the number of re-hospitalisations, to determine the interaction (group x time) effects of treatment across time. Helmert’s contrasts tests would be performed to examine any significant between-group differences on individual outcomes at each of the three post-tests (T1 to T3) if significant ANOVA results were found. Bonferroni’s corrections were adopted to adjust level of significance for multiple ANOVA analyses (i.e., adjusted *p* value = 0.01) [26]; otherwise, the level of significance of other statistical tests was set at 0.05. The AT attendance, study attrition, attendance to other psychosocial interventions, and psychotropic medications used over the study period were recorded and/or calculated.

## Results

### Sample characteristics

Of the 134 participants, 128 were included in the final data analysis (attrition rate = 4.5 %), due to lost to follow-up at T1 (*n* = 2 in TAU), mortality (*n* = 1 in TAU) and withdrawn from study participation (*n* = 2 in both AT and TAU). Three participants in AT failed to attend >3 group sessions and the mean and median attendance to AT sessions were 4.6 (SD = 1.1) and 5.0 (range 2–6), respectively.

Demographic and clinical characteristics of the 134 participants (Table 2) did not show any statistical differences between the two groups (*p* > 0.10). There were also no statistically significant differences on these characteristics between these participants and the non-participants (*n* = 266) who verbally agreed to participate (*p* > 0.20). Majority of them in AT and TAU (*n* = 57, 85 % and *n* = 56, 84 %, respectively) were taking a medium or high dosage of oral anti-psychotic medication [haloperidol equivalent mean values [19] were 9.1 mg/day (SD = 4.1) and 10.3 mg/day (SD = 5.3), respectively]; whereas, two-thirds were taking atypical and/or blended mode of anti-psychotics (*n* = 45 and 47; 67 and 70 %). Their average duration of illness was around 28 months (range 3–36 months). More than half (*n* = 36 in AT and 38 in TAU) were living with their family members and most were deemed totally non-adherence or poorly adherence to medication (*n* = 57, 85 % in AT and *n* = 60, 90 % in TAU).

**Table 2:** Demographic and clinical characteristics of participants at baseline (*N* = 134)

Characteristics		AT (*n* = 67) f (%)	TAU (*n* = 67) f (%)	*χ* ^*2*^, *P*
Gender	Male	35 (52.24)	36 (53.73)	1.25, 0.25
	Female	32 (47.76)	31 (46.27)	
Age	m*ean, s.d.*	*29.13, 9.87*	*28.23, 9.23*	
	18–29	23 (34.33)	22 (32.84)	1.32, 0.23
	30–39	31 (46.27)	32 (47.76)	
	40–49	10 (14.93)	9 (13.43)	
	50 or above	3 (4.48)	4 (5.97)	
Diagnosis	Schizophrenia	39 (58.21)	41 (61.19)	1.40, 0.21
	Other psychotic disorders	28 (41.79)	26 (38.81)	
Employment status	Employed (Full-time)	28 (41.79)	24 (35.82)	1.52, 0.17
	Employed (Part-time)	17 (25.37)	19 (28.36)	
	Unemployed	17 (25.37)	18 (26.87)	
	Others (e.g., intermittent job)	5 (7.46)	6 (8.96)	
Education level	Primary school	13 (19.40)	12 (17.91)	1.12, 0.28
	Secondary school	44 (65.67)	42 (62.67)	
	University/College	10 (14.93)	13 (19.40)	
Monthly household	*mean, s.d.*	17,915, 6,594	16,880, 6,901	1.30, 0.22
income (HK$)^a^	<10,000	9 (13.43)	8 (11.94)	
	10,001–20,000	31 (46.27)	30 (44.78)	
	20,001–30,000	21 (31.34)	21 (31.34)	
	>30,000	6 (8.96)	8 (11.94)	
Duration of illness (months)	*mean, s.d., range*	*27.71, 10.10,* *3*–*36 months*	*28.12, 10.08,* *4*–*36 months*	
	<6	18 (26.87)	20 (29.85)	1.19, 0.26
	6–12	19 (28.35)	18 (26.87)	
	13–24	18 (26.87)	16 (23.88)	
	25–36	12 (17.91)	13 (19.40)	
Treatment setting	Outpatient department	66 (98.50)	67 (100.00)	1.87, 0.13
(other than CPNS)	Day Hospital/Centre	10 (14.93)	11 (16.42)	
	Social welfare and finance	45 (67.17)	48 (71.64)	
	Individual/family counselling	14 (20.90)	12 (17.91)	
	Others (e.g., recreational and social activities and crisis intervention)	12 (17.91)	14 (20.90)	
Type of medication	Conventional antipsychotics	22 (32.84)	20 (29.85)	1.13, 0.28
	Atypical antipsychotics	30 (44.78)	32 (47.76)	
	Blended mode^c^	15 (22.39)	15 (22.39)	
Dosage of medication^b^	High	15 (22.39)	13 (19.40)	1.19, 0.27
	Medium	42 (62.69)	43 (64.18)	
	Low	10 (14.92)	11 (16.42)	
Accommodation	Private household	22 (32.84)	24 (35.82)	1.83, 0.11
	Public housing	31 (46.27)	30 (44.78)	
	Others (e.g., compassionate and long-stay care)	14 (20.90)	13 (19.40)	

*AT* Adherence Therapy, *TAU* Treatment as usual

^a^US$1 = HK$7.8

^b^Patients were taking more than one type of psychotropic medication such as the use of either conventional and atypical antipsychotics, or atypical antipsychotics and anti-depressants or anxiolytics

^c^Dosage levels of anti-psychotic medications were compared with the average dosage of medication taken by schizophrenic patients in haloperidol-equivalent mean values [19]

There were no significant differences on the baseline mean values of the outcome measures (ITAQ, PANSS, SLOF, ARS, ITAQ, and frequency and duration of re-hospitalisations) and the DAI between two groups [t(133) = 1.23–1.81, *p* > 0.12), and between two CPNS [t(132) = 1.20–1.98, *p* > 0.10], indicating no-covariant effects. There were about 1.6 times (SD = 1.5) of psychiatric hospitalisations over the past 4 months in both AT and TAU; from which, around 60 % were under ‘compulsory’ admission (66 and 67 %, respectively). There were no statistically significant differences in the amount and types of atypical versus conventional anti-psychotics (and other psychotropic drugs) between two groups at T0-T3 (using ANOVA or Chi-square test, *p* > 0.20).

### Treatment effects of adherence therapy

**Fig. 2:**
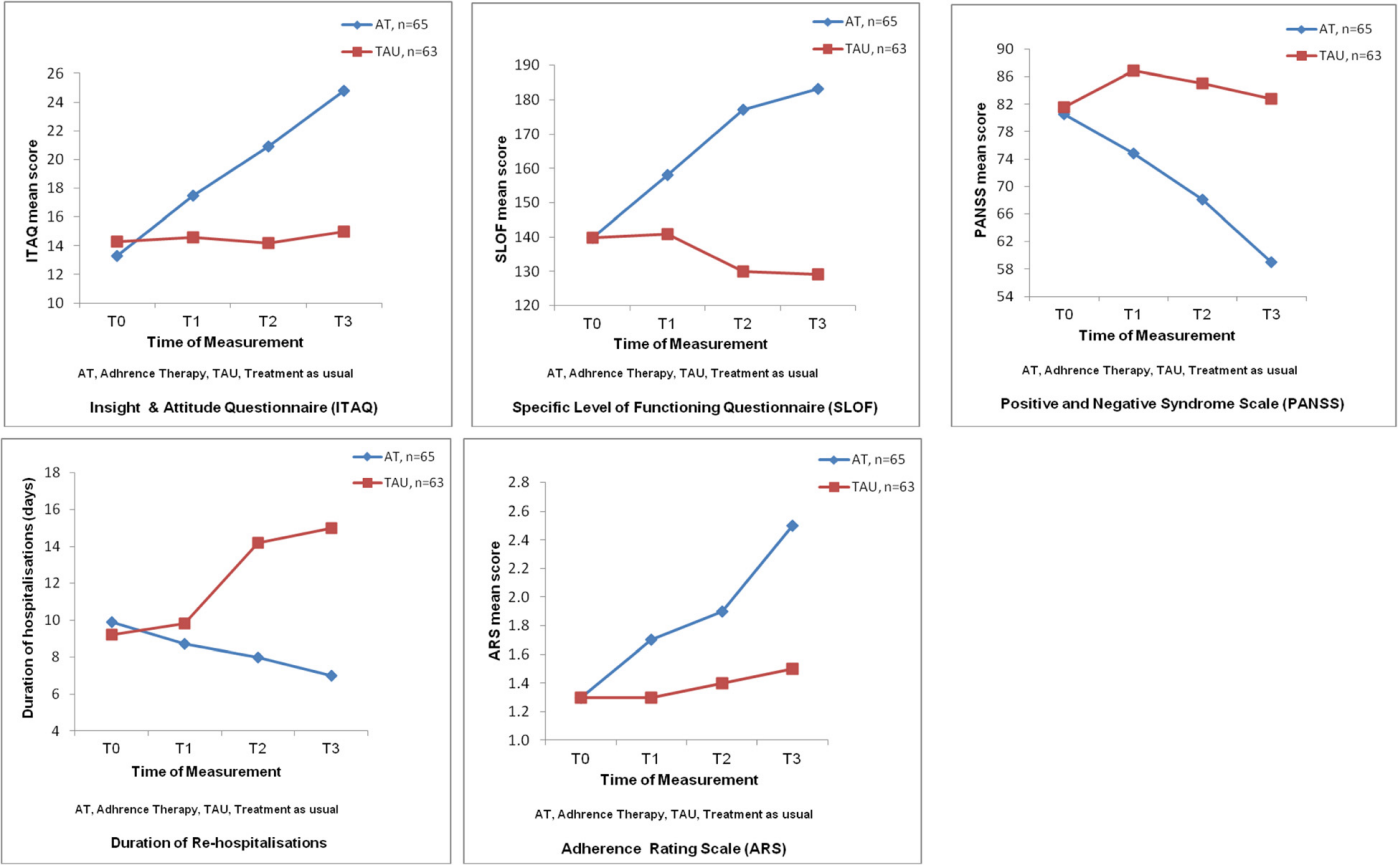
Five figures show the mean scores of each of the five study outcomes, including symptom severity (PANSS score), level of functioning (SLOF score), insight into illness/treatment (ITAQ score), duration of re-hospitalisations, for two study groups at baseline (T0) and 2 weeks, 6 months and 18 months follow-ups (T1-T3). These five study outcomes were found significantly greater improvements among the participants in adherence therapy, when compared to those in treatment as usual

The results of the outcome measures at baseline and three post-tests in both AT and TAU are summarised in Table 3. As indicated in Table 3 (using a Bonferroni’s adjusted alpha level of 0.01), there were statistically significant interaction (group x time) treatment effects of AT, which included: improvements in both the insight into illness/treatment [ITAQ score, *F*(1127) = 10.98, *p* < 0.001, Wilks’ λ = 0.35, partial η^2^ = 0.40] and functioning [SLOF score, *F*(1127) = 8.90, *p* < 0.005, Wilks’ λ = 0.30, partial η^2^ = 0.29], and reductions in symptom severity [PANSS score, *F*(1127) = 10.10, *p* < 0.003, Wilks’ λ = 0.33, partial η^2^ = 0.32] and duration of re-hospitalisations [*F*(1127) = 8.80, *p* < 0.005, Wilks’ λ = 0.29, partial η^2^ = 0.28], when compared with TAU. In addition, the medication adherence rate of the participants in AT significantly greater improved over time [ARS score, *F*(1127) = 9.10, *p* < 0.005, Wilks’ λ = 0.32, partial η^2^ = 0.30], when compared with those in TAU. An examination of the adjusted mean scores at T0-T3 (Fig. 2) indicated that the AT group reported much very consistently positive improvements in ITAQ, SLOF and PANSS scores and duration of re-hospitalisations; whereas, the TAU group indicated progressive mild to moderate negative changes of mean scores in most of their outcome scores over the 18-month follow-up. Although the average number of re-hospitalisations at T1 – T3 did not indicate any significant difference between the two study groups (H = 3.47, df = 3, *p* = 0.092), the total numbers of participants in the AT group who had at least one hospitalisation had greater and more consistent reductions from T1 to T3, when compared with the TAU group; that is, 48 (74 %) and 48 (76 %) at T1, 34 (52 %) and 50 (79 %) at T2, and 31 (48 %) and 46 (73 %) at T3, accordingly. At T0-T3 measurements, the participants’ number of re-hospitalisations ranged 0–3 times for AT and 0–4 times for TAU; whereas, its median values for AT reduced from 2 to 1 and for TAU, maintained at 2.

**Table 3:** Outcome measure scores at T0-T3 and results of repeated-measures ANOVA and Kruskal-Wallis tests for two study groups

	AT (*n* = 65)		TAU (*n* = 63)		F(1,127)	P	Effect size (Partial η^2^)
Study Outcome	Mean	s.d.	Mean	s.d.			
ITAQ (0 – 33) ^a^							
T0	13.3	3.5	14.3	5.0	**10.98**	**0.001**	0.40
T1	17.5	5.7	14.6	4.9			
T2	20.9	6.1	14.2	7.5			
T3	24.8	5.3	15.0	8.8			
SLOF (43–215)							
T0	139.8	14.1	139.8	15.1	**8.90**	**0.005**	0.29
T1	158.0	20.8	140.8	22.0			
T2	177.2	22.0	130.1	24.3			
T3	183.2	20.1	129.1	28.8			
PANSS (30–210)							
T0	80.6	7.5	81.6	6.9	**10.10**	**0.003**	0.32
T1	74.8	7.0	86.9	8.8			
T2	68.1	8.9	85.0	9.9			
T3	59.0	10.1	82.8	11.6			
ARS (1 – 5)							
T0	1.3	1.1	1.3	1.1	**9.10**	**0.005**	0.30
T1	1.7	1.0	1.3	1.1			
T2	1.9	1.1	1.4	1.2			
T3	2.5	1.3	1.5	1.1			
Re-hospitalisation							
*Frequency* ^*b*^							
T0	2 ^*c*^	(0–3) ^*c*^	2 ^*c*^	(0–3) ^*c*^	3.47 ^d^	0.092	
T1	2	(0–3)	2	(0–4)			
T2	1	(0–2)	2	(0–3)			
T3	1	(0–2)	2	(0–3)			
*Duration* ^*e*^							
T0	9.9	5.3	9.2	6.1	**8.80**	**0.005**	0.28
T1	8.7	5.1	9.8	6.8			
T2	8.0	6.6	14.2	8.2			
T3	7.0	6.0	15.0	9.9			

*AT* Adherence Therapy, *TAU* Treatment as Usual or Psychiatric Outpatient Care

*T0* baseline measurement at the start of intervention, *T1* 2 weeks after intervention, *T2* 6 months after intervention, *T3* 18 months after intervention

*ARS* Adherence Rating Scale, *ITAQ* Insight and Treatment Attitude Questionnaire, *PANSS* Positive and Negative Syndrome Scale *SLOF* Specific Level of Functioning scale

^a^ Possible range of scores of each scale indicated in parenthesis

^b^ Average number of re-admissions to a psychiatric inpatient unit in the previous 4 months at T0-T3

^c^ Median and range of the frequency of re-hospitalisations

^d^ H value of Kruskal-Wallis test (df = 3)

^e^ Duration or length of readmissions to a psychiatric inpatient unit in terms of average number of days of hospital-stay over the previous 4 months at four time points

The test (Chi-square or t) and p values are in bold if the subject characteristics are significantly different between groups

**Fig. 3:**
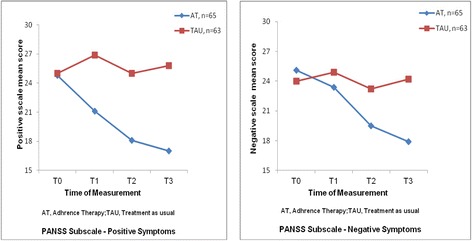
Two figures shows the mean scores of the positive and negative symptoms in PANSS for two study groups at baseline (T0) and 2 weeks, 6 months and 18 months follow-ups (T1-T3). The mean scores of these two subscales of the PANSS were found significantly greater improvements among the participants in adherence therapy, when compared to those in treatment as usual

There were also significant statistical differences on the mean scores of the two PANSS subscales between the study groups at T1-T3 (see Fig. 3). Participants in the AT reported significantly greater improvements in both positive symptoms, F(2127) = 10.85, *p* < 0.001, and negative symptoms, F(2125) = 9.14, *p* < 0.005, with effect sizes (η^2^) of 0.38 and 0.30 (large effects) [26], respectively.

Results of Helmert’s contrasts tests indicated that when compared with TAU, AT participants had significant greater changes (improvements) at the post-tests on:

- Insight into illness/treatment (ITAQ score) significantly increased at T1-T3 (Mean differences = 2.9, 3.3 and 9.8; SE = 0.9–3.2); whereas, the TAU group indicated slight changes in their insight over time;
- Symptom severity (PANSS score) significantly reduced at T1-T3 (Mean differences = 12.1, 17.0 and 23.8; SE = 1.0–1.9) and for the TAU, it slightly increased at T1 and T2;
- Level of functioning (SLOF score) significantly increased at T2 and T3 (Mean differences = 47.1 and 54.1; SE = 2.4 and 8.7, respectively);
- Medication adherence rate (ARS score) significantly increased at T2 and T3 (Mean differences = 0.5 and 1.0; SE = 0.1 and 0.2, respectively); and
- Duration (days) of re-hospitalisations significantly reduced at T2 and T3 (Mean differences = 6.2 and 8.0; SE = 2.4 and 3.8, respectively); whereas for the TAU, it was consistently increased (from 9.2 at T0 to 15.0 at T3).

There were no significant differences in the types and doses of anti-psychotic medication and nature of admission (voluntary/compulsory), as well as the types and amount of participation in other psychosocial interventions, between the two groups across four times of measurements (using repeated-measures ANOVA test or Kruskal Wallis test, *p* > 0.15). During the study period, the AT and TAU groups received other community mental health services, mainly including social skills and employment training (*n* = 23 and 25), family support group (*n* = 14 and 12), individual or family counselling service (*n* = 10 and 15), and day hospital and social and recreational centre services (*n* = 11 and 15).

There were also no significant differences on the study outcomes between the two study groups or between the participants within the AT group in terms of the six nurse therapists (CPNs), where F values of the repeated-measures ANOVA tests ranged from 1.31 to 2.89, *p* = 0.11–0.30). Intra-class correlations of outcome measures between CPNs in each of the two study settings were similar and very small, ranging 0.001 to 0.0005, which indicated very minimal nested design effects (i.e., between the six nurse therapists) on the outcomes [27].

## Discussion

### Effects of motivational interviewing-based adherence therapy

The findings of this controlled trial provide evidence on the significant positive effects of this 6-session adherence therapy (AT) for people with schizophrenia spectrum disorders in community mental health care. The results supported the study hypotheses that when compared with those in usual psychiatric outpatient care, the participants in AT indicated much better patient outcomes (i.e., psychotic symptoms, medication adherence, insight into illness/treatment, functioning, and length of psychiatric re-hospitalisations) with large effect sizes (partial η^2^ = 0.28–0.40) [26] over 18-month follow up. These findings also suggest that adherence therapy originated from Western culture [7, 25] can be effective not only in people with addictive and behavioural problems [6, 28], but also in patients with schizophrenia and other psychotic disorders.

The AT used in this study based on motivational interviewing technique is one of few interventions attempted globally to enhance patients’ insight into their medication and/or other treatments and motivation to self-manage their medication and illness-related behaviours in people with schizophrenia spectrum disorders. This AT has demonstrated promising patient outcomes over a long-term (18 months) follow-up. Whilst recent literature on adherence therapy in Thailand and European countries can only show positive effects on psychotic patients’ medication adherence and relapse rate over <12 months follow-up [8–10], the findings in this study provide strong evidence on this promising approach to AT to significantly enhance these patients’ insight into their illness and motivation to change positively in their adherence behaviours. Therefore, it demonstrates substantive improvements in a wide variety of patient outcomes, including psychotic symptoms, duration of hospitalisations and psychosocial functioning.

Although the patients’ hospitalisation rate in terms of frequency of re-admissions in psychiatric hospital/unit at T1 – T3 did not differ between groups, the duration of re-hospitalisations of the TAU group were consistently and significantly worsened, and their symptom severity (PANSS score) and functioning (SLOF score) were gradually deteriorated, over the 18-month follow-up. High percentages of the participants in the TAU had at least one hospitalisation over the follow-up (T1-T3: 76, 79 and 73 %, accordingly), whereas, the AT group reduced from 74 % at T1 to 48 % at T3. The results in the TAU group showing gradual mild deterioration in mental state and functioning over the 18-month follow-up might reflect the cumulative effects of non-adherence to medication to these patients over time. As all the patients were within 3 years and/or first-episode of illness at recruitment and low to medium dosages of antipsychotic medications, it needs time for them to show progressive deterioration in psychotic symptoms and other clinical outcomes across time as the result of poor medication and treatment adherence. These results would support the recommendation of recent practice guidelines for the importance of ensuring accessibility and adherence to treatment in the first 3 years of psychosis [1–3].

However, it remains unanswered whether the significant improvements in patient outcomes are solely the effect of AT or a combined effect of this therapy and other psychosocial and family interventions and/or psychiatric treatments that are considered potentially therapeutic but had not yet been included in the data analysis. Further research with structured, integrated rehabilitation programme to explore the therapeutic effects of its individual components, and/or compare the effects between the components, is recommended; and one of which will be the AT used in this study.

### Fidelity of adherence therapy

The AT was conducted by six trained community psychiatric nurses (three from each of the two CPNS under study) who received two full-day training, together with supervised practices on four patients, according to the AT instructor programme. However, it was guided by a validated manual and delivered by these six nurse therapists with a high level of treatment fidelity (i.e., rated ‘competent’ in >92 % of the items in the AT manual). In contrast to recent studies of AT, there were very high completion rate (95 % versus 50–70 % in other studies) [7–10] of the intervention and on other hand, low attrition rate (4 % versus 15–30 %) [7–9], over an 1-year follow-up. This AT showed much clear benefits in terms of psychopathology and treatment insight and adherence for people with acute to approaching chronic schizophrenia (3–36 months of illness), indicating poor medication adherence and moderate levels of psychotic symptoms and functioning. The sample recruited (>70 % poorly adhered to medication at baseline) was comparable and thus representative to the largest proportion of psychotic patients found during the first few years of illness [20, 29]; this has also responded to the limitation of recent AT studies in which most participants reported mildly/moderately impaired in medication adherence [5, 9, 10].

### Strengths of adherence therapy

This is one of very few clinical trials of effective adherence therapy currently available for improving patients’ insights into and adherence to anti-psychotic medication in schizophrenia and showing benefits in a variety of patient outcomes such as psychotic symptoms, re-hospitalisation rates and psychosocial functioning. This effective intervention is particularly important to these patients who would have a high relapse or recurrence rate (70–90 %) over the first few years after discharge from hospital or acute stage of illness [23, 30]. It is also worthwhile to note that this AT can improve not only these patients’ positive symptoms (e.g., hallucination and delusion) [7, 8, 10] but also treatment-resisted negative symptoms (e.g., amotivation, anhedonia and socially withdrawn). This result could be due to the effect of motivational interviewing technique in which the participants were facilitated non-judgementally to explore and resolve ambivalences on management of their illness, treatment and life problems and engaged with their intrinsic motivation to change treatment/illness-related behaviours such as medication adherence and self-care [28]. Genuine empathy, acceptance and envisioning for a better future in motivational interviewing can be helpful to access motivation and foster therapeutic growth and change specific behaviours regarding those negative symptoms [10, 28].

Several studies reported an oversight of the impact of clinicians’ characteristics in treatment effectiveness, recommending that further studies utilise patient-centred working staff who were already part of the patient’s clinical team [2, 16, 31]. The AT in this study was administered by the community psychiatric nurses of the randomly selected participants after baseline measurements had been taken. This AT were also modified to more focus on participant involvement during its second half or third phase (3 sessions), echoing the importance of enhancing a sense of self-control for the success in treatment, as suggested in studies of psychosocial interventions [2, 23, 28].

Recent reviews on AT noticed that effects of AT decay over time, or find that therapy over a course of 5–6 sessions was insufficient to be effective [7, 16, 31]. Importantly, our results can conclude a sustained effect across most outcome measures, with the observation of significantly reduced re-hospitalisations at the 18-month follow-up. Previous findings suggested that the effects of adherence therapy targeting at patients’ beliefs and insights into their illness/treatments seemed to be inconclusive [6, 9, 25]; whereas, this study confirms that such approach can be effective to outpatients with moderate psychotic symptoms in the early stage of schizophrenia spectrum disorders. The findings also echo the recommendations by Gray et al. [31] that adherence modifying factors essentially centred on improved self-determination, patient choice, and shared decision making. Further, AT is able to embody the advantages of continuous boosting up patient’s motivation to manage barriers to medication/treatment on top of essential psychosocial support and psychopharmacological education. This therapy can also acknowledge the utmost importance of patient involvement in treatment decisions, understanding about their treatments/medications used, and possible consequences of adherence or non-adherence to medication such as risk of relapse [2, 32], thus allowing patients to decide with fully informed, voluntary choices in taking their medication.

An investigation of not only individual patients’ perceptions of and satisfaction with the AT but also the therapeutic process, in terms of motivators and challenges and degrees of engagement and involvement [23], are essential to better understand the active ingredients of a motivation-enhancing behavioural intervention. Further investigation of the relationships between the perceived benefits/strengths of AT and its major components and therapeutic mechanisms can be performed using individual/group interviews and/or structured observations [9, 16]. Finally, with a longer-term follow-up (e.g., 24 months), other sustained benefits or outcomes used in psychosocial intervention studies such as improvement in global functioning, satisfaction with mental health services and quality of life, as well as its cost-effectiveness, can be investigated.

### Limitations of the study

A few limitations are noted in this study. First, over 60 % of the patients in the two CPNS were found not eligible to or excluded from the study, although the refusal rate was very low (4.5 %) when approached 650 patients for study participation. These non-participants might not be similar to the participants and their responses to the AT in this study, thus reducing the generalisability of the findings. In addition, the patients who were volunteered to participate were mainly those with full- or part-time employment (64–67 %), relatively shorter duration of illness (>55 % having less than 1 year of illness) and satisfactory accommodation and family support, and hence might be highly motivated to attend the six AT sessions (mean = 4.6 sessions, SD = 1.1). The patterns of socio-demographic and illness characteristics and levels of medication adherence or motivation to change might also not be representative to other Chinese populations with psychotic disorders. Therefore, this selective sampling should be cautioned when comparisons are made between this and other studies of medication management programmes.

Second, all of the outcome measures were reported by the participants, except the level of adherence rated by the research assistant and community psychiatric nurse. There were no biological assays such as hair, urine or blood specimens, which are considered more objective and reliable measurements [8, 10, 31], to validate or confirm with the non-invasive ones in this study. Third, multi-level modelling of data should have been performed for repeated-measures outcome analysis in this study to reduce the errors or problems with ANOVA test on sphericity assumption (constant variances of difference scores), nested design effect (sampling hierarchy in relation to more than one nurse therapists and settings used), and requirements for complete designs and data sets (or very few missing data) [33]. Last, the participants and nurse therapists were not blind to the intervention taken and Hawthorne effect (e.g., preconceived benefits of AT) among them could not be excluded.

## Conclusions

AT can be a systematic, multifaceted and client-centred therapy model with evidenced benefits and applicability to outpatients with schizophrenia spectrum disorders in a Chinese population. This therapy can also be provided by other mental health professionals if appropriately trained and become an integral part of the community-based rehabilitation programme provided by outpatient care service, together with pharmacological and other psychiatric treatments. In view of this motivational interviewing-based AT being shown effective in Chinese patients with schizophrenia, it deserves further research in the suitability for more representative samples and wider and more systematic implementation in community mental health care for patients with diverse characteristics, levels of medication adherence and illness groups.

## Abbreviations

ARS: Adherence Rating Scale
AT: Adherence Therapy
CONSORT: Consolidated Standards of Reporting Trials
CPNS: Community Psychiatric Nursing Service
CPN: community psychiatric nurse
DAI: Drug Attitude Inventory
ITAQ: Insight and Treatment Attitudes Questionnaire
PANSS: Positive and Negative Syndrome Scale
SLOF: Specific Level of Functioning Scale TAU, Treatment as usual
